# Optical imaging of subcellular fluctuations within hair cells

**DOI:** 10.1016/j.bpr.2026.100253

**Published:** 2026-01-27

**Authors:** Martín A. Toderi, Dzmitry Vaido, Dolores Bozovic

**Affiliations:** 1 Department of Physics & Astronomy, University of California, Los Angeles, Los Angeles, California; 2 California NanoSystems Institute, University of California, Los Angeles, Los Angeles, California

## Abstract

Although the transduction process has been well studied in hair cells, the possible presence of mechanical perturbations in the hair cell soma has not been explored in nonmammalian species. Hair cell mechanotransduction involves rapid biophysical events that remain difficult to observe in intact tissue. We developed a label-free optical method to image active motility within the soma during both spontaneous and mechanically driven hair bundle motion. Localized light-intensity fluctuations were detected at distinct focal planes, particularly near the periphery and basal pole of the soma. These optical signals exhibited spectral components that matched those of the hair bundle and were substantially reduced when mechanotransduction channel gating was disrupted, indicating that the somatic activity reflects physiological processes linked to mechanotransduction. Activity hotspots consistently aligned with regions of ionic channels and synaptic contacts, and strong stimulation produced phase-locked somatic responses that diminished after tip-link disruption. These findings parallel reports of mechanical correlates of neuronal activity and support the presence of an optical signature of transduction within the soma. Our results demonstrate that wide-field, label-free imaging can resolve intrinsic optical events in semi-intact sensory epithelia, offering a promising approach for noninvasive studies of hair cell and afferent-neuron signaling.

## Why it matters

Hair cells convert mechanical vibrations into the electrical signals that underlie hearing and balance, yet many of the rapid processes inside these cells remain hidden because they are difficult to measure without disrupting them. This study introduces a noninvasive optical method that detects optical changes in hair cells as they perform mechanotransduction. By revealing an optical signature of the transduction process itself, this work provides a new window into how sensory cells function. This approach could ultimately enable label-free monitoring of activity in hair cells and their connected neurons, opening new avenues for studying information flow in the auditory system.

## Introduction

Balance and audition would not be possible without the function performed by hair cells (HCs), specialized cells located in the sensory organs within the inner ear (1). In both the auditory and vestibular systems, HCs function as highly sensitive mechanical receptors, capable of detecting displacements of the hair bundle (HB) on the scale of subnanometers.

The detection of mechanical input by HCs and its conversion to electrical signals that can be transmitted to the brain involves a series of cellular events (2). Air- or ground-borne vibrations deflect the hair bundle, an organelle located at the apex of the HC soma and composed of rod-like projections, called stereocilia (3). When the hair bundle deflects, the stereocilia pivot at their base, thereby increasing the tension on the tip links that connect them. This tension opens mechano-sensitive transduction channels, allowing ionic currents to flow into the HC. The resulting depolarization prompts neurotransmitter release at the afferent synapse, generating spike trains in the neurons that relay signals to the brain (4). Conversely, efferent neurons transmit feedback signals from higher-order auditory centers back to the sensory epithelium, ensuring protective responses (5,6). Both sets of innervating fibers form synaptic boutons near the HCs’ basal pole.

In addition to mechanoelectrical transduction, the auditory system features an energy-expending active amplifier that enhances its responses to weak signals. Internal adaptation, driven by myosin motor activity, as well as calcium feedback processes that affect the opening probability of transduction channels have been shown to interact in a manner that elicits spontaneous oscillations that are innate to the hair bundle when freestanding (7). Although extensive research has characterized active motion of the hair bundle (8,9), mechanical dynamics within the soma remain largely unexplored, particularly in nonmammalian species. These HCs lack the rapid, large-scale electromotility seen in specialized outer HCs of the mammalian cochlea; however, they do exhibit related phenomena (10,11). Furthermore, intracellular processes such as ionic currents, membrane potential changes, and other biochemical transformations may elicit subtle mechanical responses (12). These potentially correlated motions within the soma could represent an overlooked component of the overall cellular response.

Prior electrophysiological studies have shown that innate mechanical oscillations of the hair bundle lead to corresponding oscillations in the transduction currents entering the soma (13). In this study, we investigate whether such biochemical and electrical activity within the soma gives rise to a measurable mechanical correlate. We developed a technique that applies wide-field microscopy and light intensity fluctuation analysis to examine optical disturbances within the HC soma. Our goal is to establish a noninvasive, label-free methodology to study HC somatic activity while minimizing disruption to their natural conditions. Our findings reveal localized hotspots within the HC soma that exhibit fluctuations linked to hair bundle motility. This technique therefore provides a valuable method for recording local mechanical perturbations at a subcellular level.

## Materials and methods

HCs obtained from dissected sacculi of the American bullfrog were imaged *ex vivo* while artificially preserving the physiological separation of the solutions bathing the epithelium. Experiments were conducted during spontaneous oscillations of the hair bundle as well as under mechanical stimulation of the stereocilia. Optical imaging techniques were employed to assess variations in light intensity corresponding to the optical fluctuations due to local changes in the tissue.

**Figure 1:**
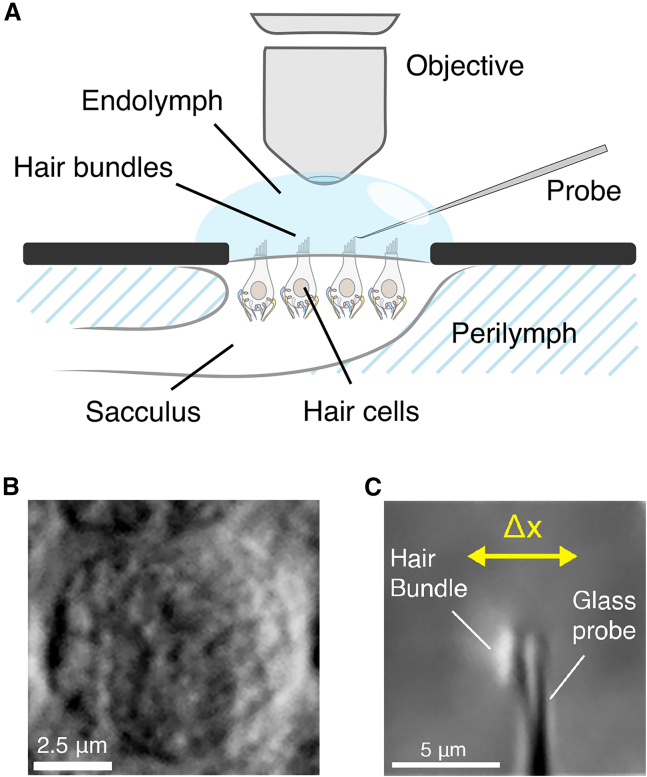
(*A*) HCs in the two-compartment chamber emulating the ionic concentrations of the natural fluid environment of the sacculus. The nerves and HC somata are immersed in perilymph in the bottom compartment and the hair bundles in endolymph at the top. Cells were accessed from the top for mechanical stimulation by the glass probes. (*B*) Bright-field image of the HC soma, 60× objective, at a selected cross section along the *z*-axis. (*C*) Image of a single hair bundle with a glass fiber attached. The focal plane is set at the tip of the stereocilia and the point of attachment of the probe. The introduced displacement Δ*x* is perpendicular to the tip of the probe.

Frogs of either gender were anesthetized (pentobarbital: 150 mg/kg), double pithed, and beheaded following protocols approved by the University of California, Los Angeles, Chancellor’s Animals Research Committee. Sacculi were excised from the inner ears of the animals, glued using the tissue adhesive Histoacryl (B. Braun, Berlin, Germany), and placed in oxygenated artificial perilymph solution (in mM as follows: 110 Na^+^, 2 K^+^, 1.5 Ca^2+^, 113 Cl^−^, 3 D-(+)-glucose, 1 Na^+^ pyruvate, 1 creatine, and 5 HEPES). The otolithic membrane was carefully removed from the epithelium after an 8-min enzymatic dissociation with 15 g/ml collagenase IV (Sigma-Aldrich, St. Louis, Missouri). This allowed mechanical access to freestanding hair bundles, which exhibited spontaneous oscillations. The epithelium was mounted in a two-compartment chamber. In this arrangement, apical surfaces were bathed in artificial endolymph (in mM as follows: 2 Na^+^, 118 K^+^, 0.25 Ca^2+^, 118 Cl^−^, 3 D-(+)-glucose, and 5 HEPES) and basolateral membranes in perilymph (Fig. 1 *A*).

HCs were treated in two different experiments with BAPTA and salicylate. BAPTA is a fast-acting calcium chelator. Replacing a 5 mM solution of BAPTA (tetrasodium salt, Millipore, Burlington, Massachusetts) in endolymph in the upper compartment of the chamber severed tip links, as calcium is crucial for maintaining the integrity of cadherin-based connections that form their structure. This process physically impairs the mechanotransduction apparatus by disrupting channel gating (14). Salicylate acts as a reversible antagonist of prestin, the motor protein responsible for outer HC electromotility in the cochlea, binding to it and suppressing its function (15). We exposed the basolateral membranes of the HCs to a 10 mM solution of Na+ salicylate (Sigma-Aldrich) in perilymph through the bottom compartment of the chamber. For both fluid exchanges, we waited 5 min to allow perfusion.

### Data acquisition

High-speed images in 16-bit TIFF format, with a resolution of 108.3 nm/px, were captured using a digital CMOS camera (ORCA-Flash4.0) at a rate of 1000 frames per second. The recordings were obtained utilizing an upright optical microscope (Olympus BX51WI) equipped with a water-immersion objective (Olympus LUMPlanFL N 60×, NA: 1.00). This configuration was affixed to an optical table (Technical Manufacturing, Peabody, Massachusetts) and established within an acoustically insulated chamber (Industrial Acoustics, Bronx, New York) to prevent any external disturbances from affecting the extremely sensitive HCs. The motion of the hair bundles was tracked according to previously developed methods (16), yielding time-dependent traces of the bundle position. The presence of robust innate oscillations was used as an indicator of the biological integrity of the sample.

After this, the focal plane was adjusted to capture sequential images at different positions along the *z*-axis (Fig. 1 *B*), with an approximate optical axial section of ≈1.46 *μ*m. This approach resulted in a z-stack of 2D cross-sectional images of the HC. The SD of the normalized intensity for each pixel,

(1) $$\sigma =\sqrt{E\left[{\left(I_{k}-\mu \right)}^{2}\right]}=\sqrt{\frac{1}{N}\sum_{k=1}^{N}{\left(I_{k}-\mu \right)}^{2}}\text{,}$$

was calculated along the time course of the recording, giving an “activity” heatmap based on light intensity fluctuations. The temporal mean matrix of the image sequence is defined as

(2) $$\mu =E\left[I_{k}\right]=\frac{1}{N}\sum_{k=1}^{N}I_{k}\text{,}$$

where **I**_*k*_ is the matrix representation of a frame intensity map, a 2D matrix composed of the grayscale levels of each pixel, and *k* is the frame number that assumes values from 0,1,…,*N*, with *N* being the total number of images from the sequence recorded during the experiment.

Based on this information, hotspots of activity were identified from the matrix *σ*, thereby outlining particular spatial areas of interest within the recording. After this step, a light intensity time series was derived by averaging the pixel values from the region of interest for each frame. The light intensity fluctuation was normalized to the mean background signal for each frame, as typically done in fluorescence microscopy, and expressed as Δ*I*/*I*.

### Mechanical stimulus

In addition to recordings captured during the spontaneous oscillations of the hair bundle, we also applied external perturbations designed to entrain this innate oscillation. Mechanical stimulation of the hair bundle was administered using a glass probe, created from a borosilicate glass capillary that was shaped using a micropipette puller (Sutter Instruments, Novato, California). These probes were coated with concanavalin A (Thermo Scientific, Waltham, Massachusetts), affixed to a piezoelectric actuator (Piezosystem Jena PA 4/12), and positioned to make contact with either the kinocilium or the tallest row of stereocilia (Fig. 1 *C*).

## Results

**Figure 2:**
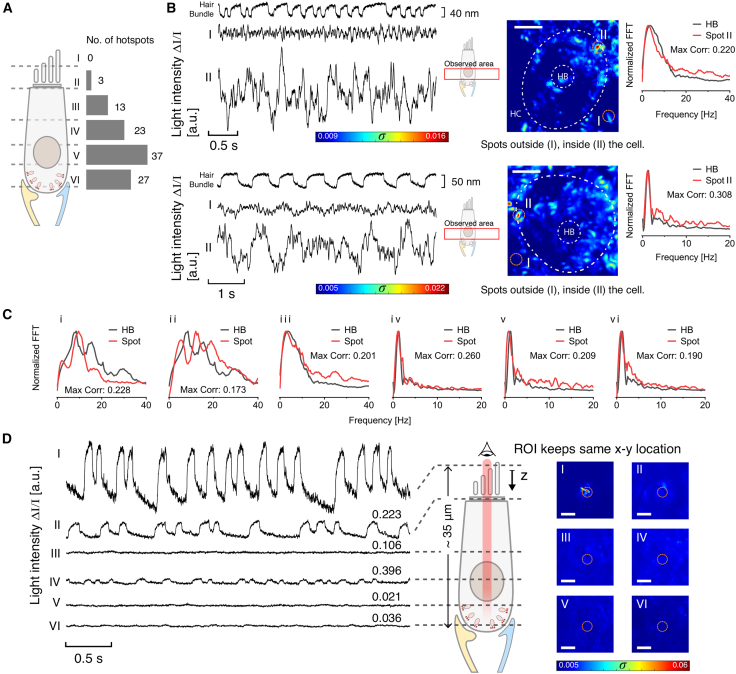
(*A*) Distribution of the number of hotspots found at different depths in the HC. (*B*) Spontaneous hair bundle motion, light traces, and *σ* heatmaps of image sequences of two HCs. Trace I corresponds to a region of interest (ROI) (*orange circle*) outside the cell, and trace II corresponds to a spot inside the cell within a hot area in the vicinity of the cell’s limits. The hair bundle projection and the HC limits are marked with white dashed lines. On the very right, the normalized fast Fourier transform (FFT) compares both hair bundle and spot II frequency spectra, annotated with the maximum correlation coefficient after all possible time shifts. (*C*) Several illustrative normalized Fourier spectra and maximum correlation coefficients for HB motion/hotspot pairs in different cells. Note that although (*ii*) and (*vi*) show maximal correlations incrementally below 0.2, they likewise exhibit similar spectra to the hair bundle motion. (*D*) Time-dependent traces of activity within the soma during innate bundle oscillations, sequentially measured at the same ROI (*orange circles on the heatmaps*) along different *z* sections. The ROI is located at the stereocilia area, and the first trace indicates its spontaneous oscillations. Traces correspond to the average light intensity of the ROI at each depth, labeled from (*I*) to (*VI*); the numbers above traces (*II*) to (*VI*) indicate the maximal Pearson correlation coefficient after optimal signal shift with respect to bundle trace I. In the cell diagram, the red stripe indicates the progression of the ROI along the *z*-axis. The rightmost heatmaps show the distribution of *σ*, keeping the same scale as for the bundle motion (*heatmap I*). The white scale bar for each heatmap denotes 2.5 *μ*m.

Our results indicate that HCs whose bundles exhibited spontaneous oscillations showed periodic fluctuations of light intensity at specific points within the somata. For our optical recordings, we compartmentalized the cell body into six axial sections to examine the locations of these innate fluctuations. The histogram in Fig. 2 *A* shows the main areas within the cell where most of the hotspots of activity were found. For 33 cells measured across six epithelia, the areas where most of the hotspots were observed were the three sections comprising the basal pole of the HC soma. As outlined in the introduction, this area is characterized by neuronal innervation. Notably, hotspots exhibited oscillatory patterns in the light trace, displaying frequencies that were comparable to those observed in the innate movement of the hair bundles. As a control experiment, we conducted comparative analyses on regions of interest both inside and outside the cell, as illustrated in Fig. 2 *B* for the same recording. The control spots, external to the cell, demonstrated no activity. The light intensity traces of hotspots within the cell body and the motion of the corresponding hair bundle yielded comparable frequency spectra, indicating similar dominant frequencies in the signals (Fig. 2 *C*). To quantify the similarity of the optical fluctuations and bundle oscillations, we computed the maximal correlation between pairs of traces after all possible time shifts. In a prior study (17), we calculated threshold values of correlation between hair bundles close to 0.2, about 4 SDs from the average distribution (approximately 0.18), thus imposing a conservative limit. A number of hotspots exhibited correlation coefficients at or above this value. These results indicate the presence of optical fluctuations within the HC somata that correlate with innate hair bundle oscillations.

To investigate how the active motility of the hair bundle affects areas situated deeper within the soma, we examined multiple successive focal planes to capture the full z-stack spanning the HC. First, we recorded hair bundle movement at the tips of the stereocilia. In each subsequent recording, we captured the light intensity traces, readjusting the focus to a new position along the *z*-axis while maintaining the *x*-*y* location of the region of interest selected from the first focal plane (Fig. 2 *D*). The recordings obtained from planes deeper within the tissue exhibited less intense light fluctuations. As these recordings were obtained sequentially as opposed to simultaneously, the correlation coefficients shown in Fig. 2 *D* are the maximum values of a set of comparisons with all possible time shifts of the bundle motion and the light trace. Trace II was recorded at the base of the pivoting stereocilia, with subsequent recordings probing regions deeper within the cell body. The maximal correlation between an oscillating light intensity trace and bundle motion was observed in the *z*-plane, approximately coinciding with the location of the cell nucleus (see Fig. 2 *C*, *trace IV*). This observation suggests that localized mechanical fluctuations within the soma occur alongside hair bundle oscillation. Although the source of this internal motion is not known, prior work has suggested that changes of the internal electric field caused by mechanoelectrical transduction could drive the motion of charged organelles (18).

**Figure 3:**
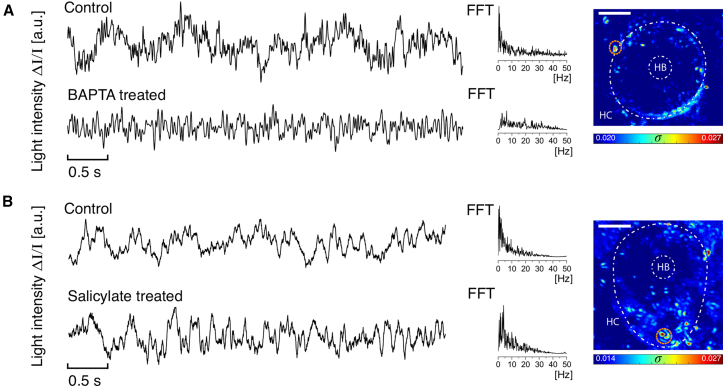
Light traces and *σ* heatmaps of deep image sequences of HCs with spontaneous oscillations of the hair bundle. The hair bundle projection and the HC limits are marked in white dashed lines. The white scale bar for each heatmap denotes 2.5 *μ*m. (*A*) The observed imaging plane was *z*-centered near the lower half of the cell. Both traces correspond to the same hotspot location (*orange circle*) detected in the control experiment and later treated with 5 mM BAPTA, which irreversibly severs the tip links, impairing mechanotransduction. The frequency spectra differed, indicating dampening of activity for the BAPTA-treated HC. (*B*) Hotspot exhibiting activity for a control HC and maintaining the trend after treatment with 10 mM Na+ salicylate, which inhibits the functioning of prestin. The spectra of both traces remain comparable.

To probe the role of transduction channel gating in eliciting somatic fluctuations, we exposed the hair bundles to BAPTA at a concentration of 5 mM. This pharmacological manipulation has been shown to lead to a severing of the tip links that connect the stereocilia, irreversibly impairing the mechanoelectrical transduction process. We tracked hotspots (*n* = 13) in 9 cells under control conditions and then introduced BAPTA into the endolymph solution. We observed diminished activity in all of the hotspots in cells treated with BAPTA, as well as suppression of peaks in the frequency spectra (representative trace shown in Fig. 3 *A*). The amplitude corresponding to the peak frequency in the Fourier spectrum changed by −65% ± 12% on average among all hotspots, from the control to the treated case. Next, we investigated a possible role of prestin in the somatic fluctuations. This protein, expressed at high levels in outer HCs of the mammalian cochlea, was shown to mediate electromotility of the somata. Although no such activity was observed in nonmammalian auditory organs, we tested its role in the internal somatic fluctuations reported here, as those had not been previously investigated. Hotspots were recorded along the *z*-axis in the vicinity of the outer walls of the soma for 15 cells, first under control conditions and subsequently treated with salicylate, shown to block prestin function. No notable change was observed in the light intensity fluctuations of the hotspots (*n* = 21); Fig. 3 *B* depicts the traces measured in one representative hotspot. The average amplitude change in the peak frequency of the Fourier spectrum of all the hotspots was 7% ± 5%, indicating invariance to the treatment with salicylate. Prestin activity therefore does not account for the observed mechanical fluctuations.

### Somatic activity evoked by mechanical deflections of the hair bundle

A glass probe mounted on a piezoelectric actuator enabled us to apply mechanical deflections of a specified amplitude and frequency. Micromanipulation ensured that the probe contacted only one hair bundle, with its tip attached to either the kinocilium or the row of tallest stereocilia.

**Figure 4:**
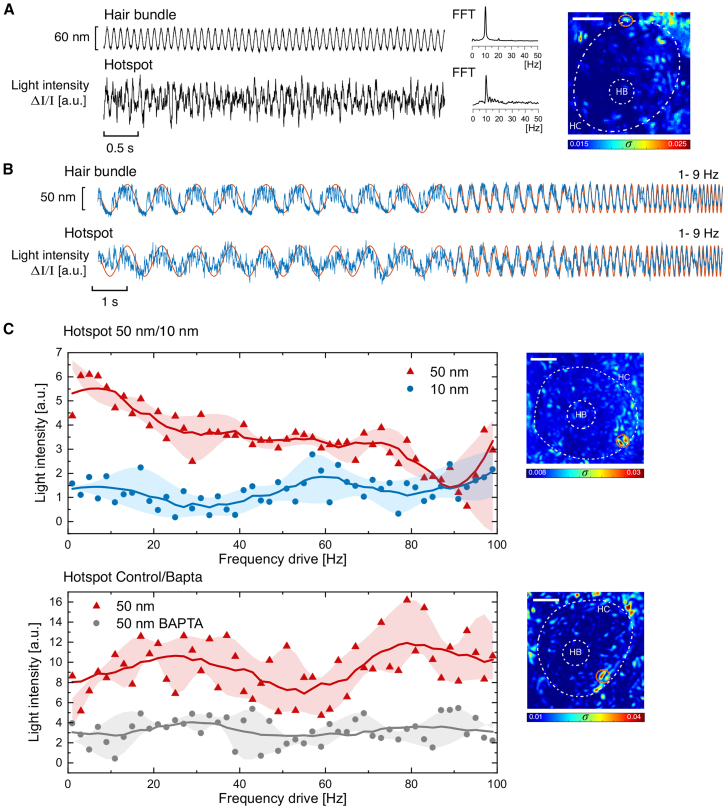
(*A*) Motion of a hair bundle driven by a glass probe attached to the tips of the stereocilia, light trace for a ≈25-*μ*m-deep hotspot, frequency spectra for both traces, and heatmap of the z slice. The probe introduced a 10 Hz sinusoidal oscillatory displacement, perpendicular to the rows of stereocillia, producing displacements of 60 nm. Most prominent hot areas were found closer to the basal pole of the cell. (*B*) Hair bundle motion trace exhibiting strong phase locking to the mechanical stimulus frequency sweep; next, a hotspot in that cell (*light intensity trace together with the stimulus in orange*). (*C*) Frequency sweep phase-locking curve for stimulating amplitudes of 50 and 10 nm of a hotspot light intensity (*shaded envelopes* indicate dispersion of values). The images on the right are heatmaps showing the outline of each cell, the projected position of the HB (*white dashed lines*), and the analyzed hotspot (*orange*). The white scale bar for each heatmap denotes 2.5 *μ*m. The bottom image shows the frequency-sweep phase-locking curve of a hotspot at a stimulating amplitude of 50 nm for a healthy HC (red) and then for an HC treated with BAPTA (gray), physically impairing mechanotransduction.

The first protocol involved applying pure-tone signals to the hair bundle and detecting resulting responses in the soma. Specifically, a 10 Hz sinusoidal stimulus inducing approximately 60 nm of hair bundle displacement was applied for 5 s. As expected, the bundle was entrained by the applied signal, adjusting its oscillations to the 10 Hz drive. Plots of *σ* obtained from deeper focal planes during the application of the stimulus yielded clear hot areas, one of which is depicted in Fig. 4 *A*. Prominent signals were detected in the lower regions of the cell body, with hotspots typically occurring near the HC edges. The frequency spectra of the hotspot traces matched those of the corresponding hair bundle oscillations (Fig. 4 *A*). Our results indicate that the motion of the hair bundle triggers mechanical fluctuations at the same frequency deep within the cell soma. We note that these observations are unlikely to be due to an optical artifact, whereby deflections of the hair bundle lead to shadowing or other optical projections onto lower layers. First, the observed somatic activity is not uniform or monotonic along the z-stack but rather shows maximal activity near the basal end of the soma. Second, the hotspots are not localized directly underneath the hair bundle but rather near the circumference of the cell body.

### Entrainment of somatic fluctuations by an applied frequency sweep

Prior studies have shown that spontaneously oscillating hair bundles can be phase locked by an applied signal. Stimuli of very weak amplitude lead to entrainment only at frequencies comparable to those of spontaneous oscillations, whereas stronger signals phase lock the innate motility over a broader bandwidth (19).

We utilized a glass probe driven by a piezoelectric actuator to apply sinusoidal displacements to spontaneously oscillating hair bundles (*n* = 9). The frequency of the signal was swept from 1 to 100 Hz at fixed amplitudes of 10 and 50 nm. For all measurements, the glass probe was selected to be sufficiently stiff so as to drive the cell at high amplitudes but flexible enough to allow spontaneous oscillations to persist upon attachment (*K*_*probe*_ = 150 *μ*N/m). The 10 nm signal served as a control, as it was observed to be too weak to elicit a response. In contrast, the 50 nm signal was consistently found to both entrain the hair bundle motion and trigger activity within the soma. The top trace in Fig. 4 *B* shows hair bundle motion, with the 50-nm-amplitude stimulus superposed; the plot shows a low range of frequencies (1–9 Hz) for visibility. Phase locking of active bundle motility is evident in the response, with sporadic spike-like excursions observed between cycles at the lower frequency end. The bottom trace shows a recording of light intensity fluctuations obtained from a somatic hotspot, which likewise exhibited appreciable phase locking. For the sake of clarity, we display only a particular frequency range, where the response is more prominent. To quantitatively assess entrainment over the full 1–100 Hz range, we define the phase-locked response as the amplitude of the displacement’s frequency component that matches that of the stimulus frequency. This amplitude was computed using the fast Fourier transform of each portion of the signal (10 cycles). The top image in Fig. 4 *C* shows the phase-locked response of a selected hotspot over the full frequency sweep, obtained for 10 and 50 nm signals. The hotspot can be observed in the outlined region within the heatmap to the right. These frequency sweeps elicited activity in hotspots manifesting broadband phase-locking curves, with a diminished response at higher frequencies. The 50-nm-amplitude frequency sweeps elicited robust phase locking, whereas the sweeps applied at 10 nm amplitude did not trigger measurable activity.

Next, we aimed to determine whether intact gating of mechanoelectrical transduction channels is required to elicit activity within the soma. We hence applied BAPTA, a pharmacological agent shown to disrupt tip links, to determine whether this manipulation eliminates the phase-locked response. Fig. 4 *C* (*bottom*) compares the phase-locked response for a hotspot in a healthy cell (shown in *red*) with that of the same hotspot recorded after treatment with BAPTA (shown in *gray*). For both sets of recordings, the hair bundle was stimulated with a frequency sweep at an amplitude of 50 nm. Our results show that physically impairing channel gating in the hair bundle eliminates the somatic activity elicited in response to an applied drive.

## Discussion

This study introduces a label-free optical method designed to image active motility within various components of an HC soma. HCs were stimulated through the mechanical deflection of their hair bundles while recording the resulting optical changes at localized spots within the cell body. Variations in light intensity were observed at distinct focal planes along the *z*-axis, specifically near the edges of the cell somata. Robust somatic activity was observed both when hair bundles were unperturbed and exhibited spontaneous oscillations and when they were driven by an attached stimulus fiber. In both cases, the light intensity fluctuations in the soma showed spectral components that reflected those of the bundle. Furthermore, the dynamics of the observed optical fluctuations diminished when the channel-gating apparatus in the bundle was impaired, suggesting a causal relationship between these phenomena. We therefore propose that the observed changes in light intensity are due to tissue perturbations associated with the mechanotransduction process.

When external signals deflect the hair bundles, the opening of the mechanically sensitive transduction channels leads to a cascade of cellular events that result in a net depolarization of the HC. Changes in the membrane potential are in turn accompanied by influx and efflux of ions, as well as a number of other internal processes (2). The refractive index of biological tissue is subject to alteration due to multiple influences, such as variations in hydration levels, vesicle and membrane dynamics, and potential physical deformations of the tissue itself (20). These modifications play a significant role in light-scattering phenomena and may lead to optical fluctuations. Although the specific mechanism of the observed somatic activity is beyond the scope of the current study, our results indicate that the cascade associated with mechanoelectrical transduction leads to measurable physiological changes in specific areas within the cell body.

As one potential mechanism that could lead to mechanical motility within the soma, we explored the role of prestin, a protein expressed within the basolateral membrane of mammalian outer HCs (21), as well as in the cells of the vestibular system (22). Recently, studies on prestin analog genes in the bullfrog’s (*Rana catesbeiana*) inner ear organs, including the sacculus, showed cells with nonlinear capacitance, a main feature of prestin motor function (11). Furthermore, transfected bullfrog’s prestin showed electrophysiological features similar to those of HCs from its inner ear (23). Although electromotility has not been observed in bullfrog sensory epithelia, we tested whether it can account for any of the activity observed in our experiments. Introduction of salicylate, which prevents prestin from transitioning into the contracted state, eliminates this possibility.

A remarkable aspect throughout these observations is the location of the activity spots within the cell soma. These elements are consistently found around the limits of the HC and in the *z*-planes near the basal pole of the cell, coinciding with the general location of ionic channels and synaptic contacts by innervating neurons. For sufficiently strong stimuli applied to the bundle, we observed phase locking of the optical fluctuations over the physiological frequency range, consistent with the known response of driven active hair bundles (19). As severing the tip links dulled this phase-locked response, depolarization of the HC and the corresponding synaptic activity may play a role in evoking the observed somatic fluctuations. In particular, a large number of experimental studies have shown that neuronal activity is accompanied by fast and temporary mechanical changes. These include changes in axonal radius (24,25), pressure (26), optical properties (27), the release and subsequent absorption of heat, and shortening of the axon at its terminus. More recently, interferometric experiments on isolated neurons indicated nanometer-scale deformations during action potentials (28). Hence, other studies have reported observations of mechanical deformations in response to electrical or chemical changes accompanying neural activity. Consistent with these observations, our results indicate that an optical correlate of the transduction process can be observed within the HC soma.

The methodology developed in these experiments provides wide-field, label-free imaging of subcellular mechanical motion. Similar applications have been explored in recordings of neural signaling by imaging mechanical correlates of synaptic activity (29). Our findings therefore extend these applications, as they show that wide-field microscopy, when applied to semi-intact sensory epithelia, provides a viable approach for tracking active internal processes within the HCs. Further development of our noninvasive imaging technique could pave the way for future applications in spike train recordings in afferent neurons, thus enabling new measurements of information flow in the auditory system.

## Data and code availability

Data sets generated during the current study are available from the corresponding author upon reasonable request.

## Acknowledgments

M.A.T. thanks Dr. Justin Faber for his help with the glass probe making. This research was funded by the US DoD - Air Force Office of Scientific Research, grant FA9550-23-1-0713.

## Author contributions

M.A.T. and D.B. conceived the project. M.A.T. and D.V. performed the experiments. M.A.T. analyzed the data. M.A.T. wrote the manuscript with input from all authors.

## Declaration of interests

The authors declare no competing interests.
